# Circulating tumor cells and palbociclib treatment in patients with ER-positive, HER2-negative advanced breast cancer: results from a translational sub-study of the TREnd trial

**DOI:** 10.1186/s13058-021-01415-w

**Published:** 2021-03-24

**Authors:** Francesca Galardi, Francesca De Luca, Chiara Biagioni, Ilenia Migliaccio, Giuseppe Curigliano, Alessandro M. Minisini, Martina Bonechi, Erica Moretti, Emanuela Risi, Amelia McCartney, Matteo Benelli, Dario Romagnoli, Silvia Cappadona, Stefano Gabellini, Cristina Guarducci, Valerio Conti, Laura Biganzoli, Angelo Di Leo, Luca Malorni

**Affiliations:** 1 “Sandro Pitigliani” Translational Research Unit, Hospital of Prato, Prato, Italy; 2 Bioinformatics Unit, Hospital of Prato, Prato, Italy; 3 Division of Early Drug Development, Istituto Europeo di Oncologia, IRCCS, Milan, Italy; 4 Department of Haematology and Haemato-Oncology, University of Milan, Milan, Italy; 5 Department of Oncology, Azienda Sanitaria Universitaria del Friuli Centrale, Udine, Italy; 6 “Sandro Pitigliani” Medical Oncology Department, Hospital of Prato, Prato, Italy; 7 School of Clinical Sciences, Monash University, Melbourne, Australia; 8 Department of Medical Oncology, Dana-Farber Cancer Institute, Boston, USA; 9 Pediatric Neurology, Neurogenetics and Neurobiology Unit and Laboratories, Children’s Hospital A. Meyer-University of Florence, Florence, Italy

**Keywords:** Liquid biopsy, CTCs, Luminal breast cancer, Metastatic, Palbociclib, CDK4/6 inhibitor, ddPCR, Biomarker

## Abstract

**Background:**

Circulating tumor cells (CTCs) are prognostic in patients with advanced breast cancer (ABC). However, no data exist about their use in patients treated with palbociclib. We analyzed the prognostic role of CTC counts in patients enrolled in the cTREnd study, a pre-planned translational sub-study of TREnd (NCT02549430), that randomized patients with ABC to palbociclib alone or palbociclib plus the endocrine therapy received in the prior line of treatment. Moreover, we evaluated *RB1* gene expression on CTCs and explored its prognostic role within the cTREnd subpopulation.

**Methods:**

Forty-six patients with ER-positive, HER2-negative ABC were analyzed. Blood samples were collected before starting palbociclib treatment (timepoint T0), after the first cycle of treatment (timepoint T1), and at disease progression (timepoint T2). CTCs were isolated and counted by CellSearch® System using the CellSearch™Epithelial Cell kit. Progression-free survival (PFS), clinical benefit (CB) during study treatment, and time to treatment failure (TTF) after study treatment were correlated with CTC counts. Samples with ≥ 5 CTCs were sorted by DEPArray system® (DA). *RB1* and *GAPDH* gene expression levels were measured by ddPCR.

**Results:**

All 46 patients were suitable for CTCs analysis. CTC count at T0 did not show significant prognostic value in terms of PFS and CB. Patients with at least one detectable CTC at T1 (*n* = 26) had a worse PFS than those with 0 CTCs (*n* = 16) (*p* = 0.02). At T1, patients with an increase of at least three CTCs showed reduced PFS compared to those with no increase (mPFS = 3 versus 9 months, (*p* = 0.004). Finally, patients with ≥ 5 CTCs at T2 (*n* = 6/23) who received chemotherapy as post-study treatment had a shorter TTF (*p* = 0.02). Gene expression data for *RB1* were obtained from 19 patients. CTCs showed heterogeneous *RB1* expression. Patients with detectable expression of *RB1* at any timepoint showed better, but not statistically significant, outcomes than those with undetectable levels.

**Conclusions:**

CTC count seems to be a promising modality in monitoring palbociclib response. Moreover, CTC count at the time of progression could predict clinical outcome post-palbociclib. *RB1* expression analysis on CTCs is feasible and may provide additional prognostic information. Results should be interpreted with caution given the small studied sample size.

**Supplementary Information:**

The online version contains supplementary material available at 10.1186/s13058-021-01415-w.

## Background

Molecular biomarkers are central to the personalization of cancer treatment. However, a given marker may be dynamic and has potential to change during tumor evolution and metastasis [1]. Liquid biopsy is a minimally invasive tool that allows monitoring of cancer evolution in real time. Several circulating biomarkers, including circulating tumor cells (CTCs), circulating tumor DNA (ctDNA) and RNA, exosomes, proteins, or metabolites, are being investigated as tools for monitoring disease progression and treatment efficacy [2].

CTCs are cancer cells that detach from primary tumor, and/or metastases, and enter in the bloodstream. Despite challenges in CTC detection due to their low concentration in the blood, the prognostic role of CTCs has been extensively demonstrated by several clinical studies [3–5]. Moreover, CTCs can provide important information about tumor formation and evolution [6]. CTCs can encompass the full spectrum of molecular changes in metastases, with advancements in technology allowing the characterization of the genomic, transcriptomic, and even proteomic status of single CTCs [7–11]. This, coupled with the option to collect CTCs in longitudinal blood samples over time, suggests great potential for CTCs as a pharmacodynamic marker for guiding real-time drug selection during disease progression.

CDK4/6 inhibitors (CDK4/6i) plus endocrine therapy are the mainstay of treatment for patients with estrogen receptor (ER)-positive, human epidermal growth factor receptor 2 (HER2)-negative advanced breast cancer (ABC). However, not all patients respond to these agents, and even patients with an initial disease response will eventually develop acquired resistance [12, 13]. Despite several efforts in identifying prognostic and predictive biomarkers of resistance to CDK4/6i [14], to date no biomarker (apart from endocrine receptor status) has been approved to select patients before treatment, or to monitor treatment efficacy. Additionally, therapeutic options after disease progression to CDK4/6i are currently chosen based on empirical considerations. These options generally consist of additional endocrine therapy +/− a biologic agent, or chemotherapy. Currently there is no biomarker to assist in stratifying patients based on individual prognosis or to guide personalized treatment selection in this setting.

cTREnd is a pre-planned translational substudy of the TREnd trial (NCT02549430) [15], with the aim to identify circulating biomarkers of palbociclib resistance. In this study, we evaluated the prognostic role of CTC count and its potential in monitoring palbociclib treatment and in predicting outcome to the line of therapy received after disease progression on TREnd trial. Furthermore, we set up a digital PCR assay to evaluate *RB1* gene expression (GE) on pure CTCs sorted by DEPArray and explore the clinical value of *RB1* positive CTCs.

## Methods

### Patients and sample collection

**Fig. 1:**
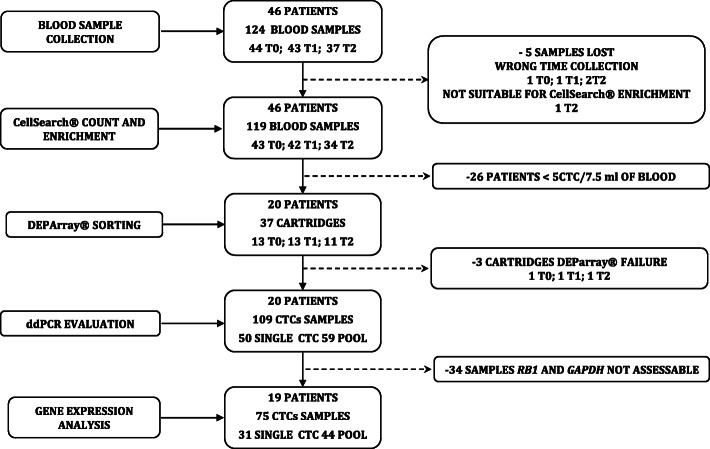
c-TREnd study flowchart

The TREnd trial was a phase II, open-label, multicenter study that randomized 115 patients with endocrine-resistant ER-positive, HER2-negative ABC to receive either oral palbociclib monotherapy, or palbociclib (at the same dose and regimen) in combination with the same endocrine therapy received in the prior line of treatment [15]. A translational sub-study, cTREnd, was conducted in parallel with TREnd, with the aim to identify potential circulating biomarkers of palbociclib resistance. Both the main study and translational sub-study gained prospective approval from the independent local ethics committees of each participating center. Patients joining the c-TREnd sub-study signed a separate informed consent. For CTC analysis, whole blood samples were collected in CellSave preservative tubes (Menarini, Silicon Bio-system, Bologna, Italy). Forty-six patients were enrolled for CTC analysis. Forty-four samples were collected at baseline, before starting trial treatment (timepoint T0); 43 samples were collected after the first cycle of treatment (T1) and 37 at the time of progression on trial or before starting a new line of therapy (T2). Matched samples from all three timepoints were available for 34 patients, while nine patients had matched samples for two timepoints, and another three had available samples from one timepoint. Only patients with five or more CTCs after CTC enrichment (*n* = 20) were sorted by DEPArray^TM^ (DA) for *RB1*-*GAPDH* gene expression (GE) quantification by droplet digital polymerase chain reaction (ddPCR). Study flow chart and numbers of available samples for each analysis are presented in Fig. 1.

### CTC enrichment and sorting

CTC enrichment and enumeration were carried out by CellSearch® System (CS) (Menarini Silicon Biosystems). CTCs were isolated from 7.5 mL of whole blood with CellSearch™Epithelial Cell kit. After enrichment, isolated and stained cells were identified and counted in the CellTracks® Analyzer II. Cartridges from patient samples with five or more CTCs were kept in the dark at 4 °C before cell sorting by DEPAarray™ System (Menarini Silicon Biosystems).

For DEPAarray sorting, stained cells in suspension were recovered from CellSearch cartridges and, after a volume reduction, were loaded into the DA A300K DS V2.0 chip (Menarini Silicon Biosystems) and set into the DA system, according to the manufacturer’s instructions. After identification, cells were manipulated by dielectrophoresis cages and recovered as single CTCs or pools of pure CTCs into 200 μL tubes. CTC samples were then stored at − 20 °C for later RNA extraction, according to the manufacturer’s protocol.

### ddPCR

To quantify CTC mRNA, we used the highly sensitive method QX200 ddPCR System (Bio-Rad, Hercules, CA). We set up a duplex ddPCR protocol to simultaneously evaluate *RB1* as the gene of interest and *GAPDH* as a reference gene for CTC presence.

The ddPCR assay was set up initially on three breast cancer cell lines (T47D, MDAMB361, and BT474) sensitive to palbociclib (PDS) and on their resistant derivatives (PDR). These cell lines—both PDS and PDR—were previously characterized for *RB1* expression by gene expression profile and Western blot experiments [16]. Growth conditions of the cell lines have been previously described [16].

RNA from cell lines was extracted by RNeasy Mini kit (Qiagen, Hilden, Germany) and retro-transcribed by iScript advanced cDNA synthesis kit (Bio-Rad). To evaluate assay sensitivity, we determined the linearity of the *RB1*/*GAPDH* titrating down cell line cDNA from 250 to 15 pg/μL. ddPCR assay was set up in a reaction mix containing PrimePCR™ ddPCR™ Expression Probe *RB1*, FAM labeled (Bio-Rad), PrimePCR™ ddPCR™ Expression Probe *GAPDH,* HEX labeled (Bio-Rad), ddPCRSupermix for Probes, no dUTP (Bio-Rad, Hercules). Droplet emulsion was obtained mixing ddPCR reaction mix with Droplet Generation Oil by QX200 Droplet Generator (Bio-Rad). Every ddPCR plate contained a triplicate of every dilution point and water as no template control. Amplification was performed at 95 °C for 10 min, followed by 40 cycles at 94 °C for 30 s and 58 °C for 1 min, with a ramp rate of 2 °C/s by T100 Thermal cycler (Bio-Rad). QX200 Droplet Reader was used to read amplified samples, and gene copy number was calculated by QuantaSoft Analysis Pro software (Bio-Rad). *RB1* and *GAPDH* expression were quantified as gene copy number/μL.

### CTC RNA extraction, cDNA synthesis, and ddPCR quantification

CTC RNA extraction and reverse-transcription were performed using Cell Lysis Two-Step RT-qPCR Kit (Bio-Rad, Hercules, CA, USA) directly into the 200 μL tubes of the DA recovered CTCs.

The entire volume was used for the ddPCR assay. Every ddPCR plate contained T47D PDS cDNA as positive control and water as no template control.

As a standard practice, no threshold was applied [17, 18], patients with ≥ 1 *RB1* or *GAPDH* copies per microliter were considered *RB1* positive or *GAPDH* positive, respectively. To analyze *RB1* and *GAPDH* expression in patients with multiple CTC samples we defined a mean copy number (MCN) calculated as the ratio between total number of gene copies and total number of CTCs.

### Statistical analysis

Progression-free survival (PFS) was estimated as the time from treatment initiation on trial to radiological disease progression or death. Time to treatment failure (TTF) was the time interval between initiation of a new therapy and its discontinuation for any reason. The distributions of PFS and TTF were estimated using the Kaplan–Meier method. Hazard ratios (HRs) with 95% confidence intervals (CIs) were calculated with the Cox proportional hazards model.

CTC count was examined as a continuous variable and as a univariate analysis using Cox proportional hazards model and categorized by one or five cell cutoffs and compared with the log-rank test. Clinical benefit (CB) was defined as the percentage sum of complete responses (CR), partial responses (PR), and stable disease (SD) for at least 24 weeks according to RECIST 1.1 criteria. Odds ratios are defined as relative odds of the occurrence of CB, given the co-occurrence of the variable of interest (CTC counts, CTC dynamic change). Wilcoxon test was used to compare mean copy number of *GAPDH* and *RB1.* Ordinary one-way ANOVA was used to compare PDS and PDR *RB1* and *GADPH* expression to evaluate assay validity. Fisher test and Runs test were used to evaluate linearity of *RB1*/*GAPDH* ratio. As exploratory analyses, the *p-*values and supportive analyses should be considered descriptive only.

## Results

### Patient characteristics

Forty-six patients were enrolled in the cTREnd study, 20 from the single-agent palbociclib arm and 26 from the combination arm. Baseline characteristics were well-balanced between the two treatment arms. In keeping with the characteristics of the patients in the overall TREnd cohort (Additional Table 1), at enrollment, most patients in the cTREnd cohort had an Eastern Cooperative Oncology Group (ECOG) performance status of 0, had evidence of visceral metastases, and had completed only one prior line of endocrine therapy in the advanced setting. Prior to entering the study, the majority of patients was on the most recent endocrine therapy for more than 180 days and had received no prior chemotherapy for metastatic disease. Similar to the TREnd trial, in the cTREnd cohort, we did not observe differences in patient’s outcome between the two treatment arms in terms of CB (Additional Table 2) and PFS (HR 0.72; 0.4–1.32 95% CI; *p* = 0.29 for palbociclib + endocrine therapy versus palbociclib alone). Median CTC count at T0 and at T1 were not significantly different between treatment arms. Given the small number of patients in cTREnd, all the analyses were performed on the entire cohort irrespectively of the treatment arm.

### CTC enrichment, enumeration by CellSearch, and clinical characteristics

Additional Table 3 illustrates the number of isolated CTCs per patient and timepoint. At least one blood sample per timepoint was analyzed for all enrolled patients. Overall CS enrichments were carried out for 43/44 T0 samples, 42/43 T1 samples, and 34/37 T2 samples. Median CTC count was 1 CTC/7.5 mL (range 0–331) at T0, 1 CTC/7.5 mL (range 0–294) at T1, and 2 CTCs/7.5 mL (range 0–317) at T2. Twenty patients had five or more CTCs. Overall, we analyzed 13/43 samples at T0 (30%), 13/42 samples at T1 (31%), and 11/34 samples at T2 (32%). Patient characteristics according to the number of CTCs at baseline are presented in Additional Table 4. Patients with less than 5 CTCs were more frequently found to have an ECOG performance status of 0 while the group of patients with baseline CTC > 0 was significantly enriched for patients who had been treated for > 6 months in the prior line of ET before the study entry (*p* = 0.04).

### CTCs count and PFS

We first analyzed the association between median progression-free survival (mPFS) and baseline (T0) CTC counts, both as a continuous variable and with cut-offs of > 0 cells/7.5 mL or ≥ 5 cells/7.5 mL. No prognostic value was found for CTC count at baseline for any of the cut-offs analyzed (*p* = 0.27, *p* = 0.78, *p* = 0.19 for the continuous variable, 0 and 5 CTC cutoffs, respectively).

**Fig. 2:**
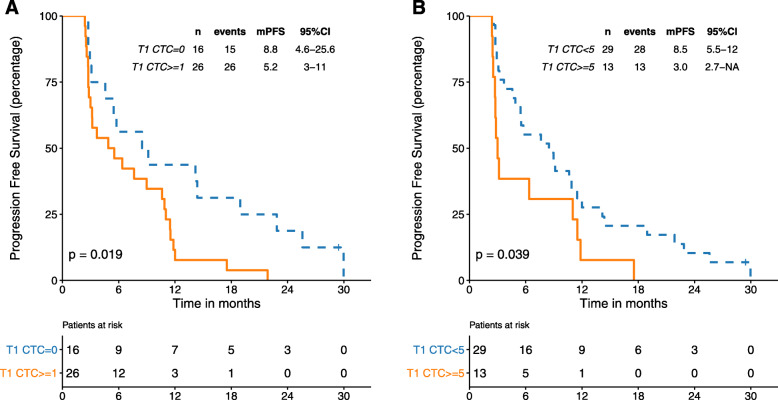
PFS according to the number of CTCs at T1 timepoint. 2: **a** CTCs categorized by 1 or more CTCs and = 0 CTCs cut off; **b** categorized by < 5 and ≥ 5 CTCs cut off

We next analyzed whether a count performed at the end of the first cycle of palbociclib (T1) could predict PFS. We found that patients with 1 or more CTCs at T1 (*n* = 26) had a significantly worse outcome compared to those with 0 CTCs (*n* = 16) (mPFS 5 vs 9 months, *p* = 0.02). Results remained significant when utilizing 5 CTCs as a cut-off. Patients with ≥5 CTCs (*n* = 13) showed a mPFS of 3.0 versus 8.5 months for patients with < 5 CTCs (*n* = 29, *p* = 0.04) (Fig. 2a, b). Concordant results were obtained when CTC count was employed as a continuous variable (*p* = 0.0002).

**Fig. 3:**
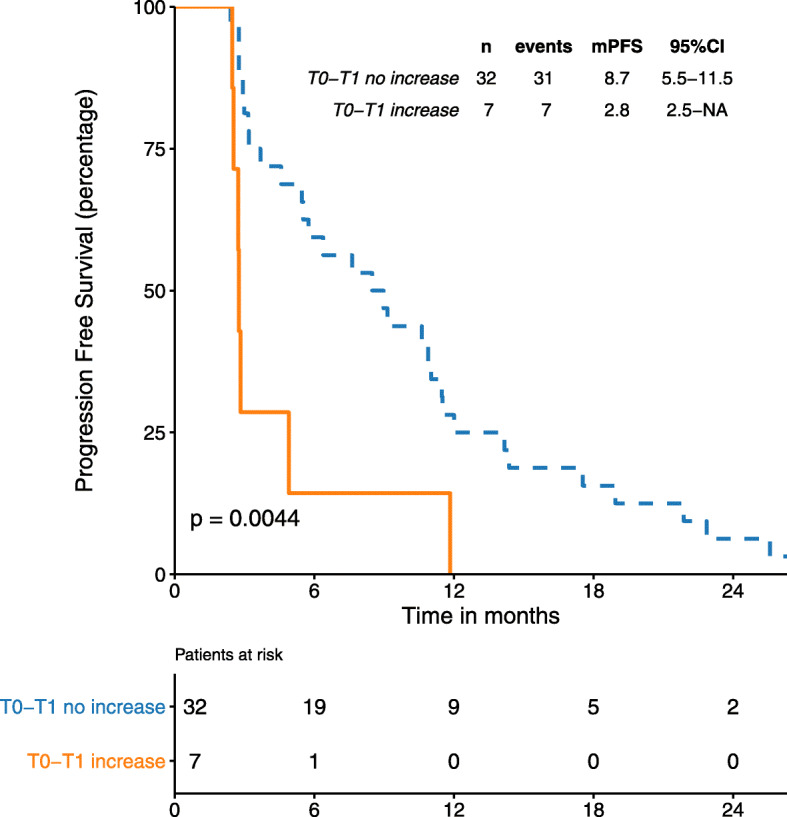
PFS according to T1-T0 change. “increase” category when nCTCs T1 – nCTCsT0 > 3, “no increase” category when nCTCs T1 – nCTCs T0 < 3

Finally, we assessed the dynamics of CTC counts between timepoints T0 and T1. Matched CTC counts at T0 and T1 were available for 39 patients. Changes in counts between the two timepoints were categorized as an “increase” when the difference between T1 and T0 ≥ 3 (*n* = 7), and “no increase” when the difference was < 3 (*n* = 32). Patients with a CTC “increase” had a mPFS of 3 months, compared to 9 months for those with “no increase” (*p* = 0.004) (Fig. 3). Significant differences (*p* = 0.01) were also observed when patients were categorized into three groups: CTC “drop” when T0–T1 ≤ 3 (*n* = 13), “increase” when T1–T0 ≥ 3 (*n* = 7), and “no change” when the difference between the two timepoints was 0 (*n* = 19). The mPFS of patients in the “increase” group was 3.0 months, compared to 8.5 and 9.0 months in the “no change” and “drop” groups, respectively.

### CTC count and clinical benefit

The association between CB and CTC count is shown in Additional Table 5. No significant associations between CB and CTCs count were observed at any timepoint. At baseline (T0), 74% of patients who achieved CB had a CTC count < 5 (OR 0.58, 95% CI 0.15–2.25, *P* = 0.424).

After 1 month of treatment (T1), 80% of patients achieving CB had < 5 CTCs, (OR 0.28; 95% CI, 0.07 to 1.07; *P* = 0.063). Finally, considering CTC change between T0 and T1, 8% of patients in the “increase” group achieved CB, versus 92% of patients in the “no increase” group (OR 5.5; 95% CI, 1 to 43.38; *P* = 0.085). A spider plot describing the dynamic changes from baseline CTC absolute count for patients with complete samples’ set is presented as additional figure 1.

### CTC count at disease progression and post-TREnd outcome

**Fig. 4:**
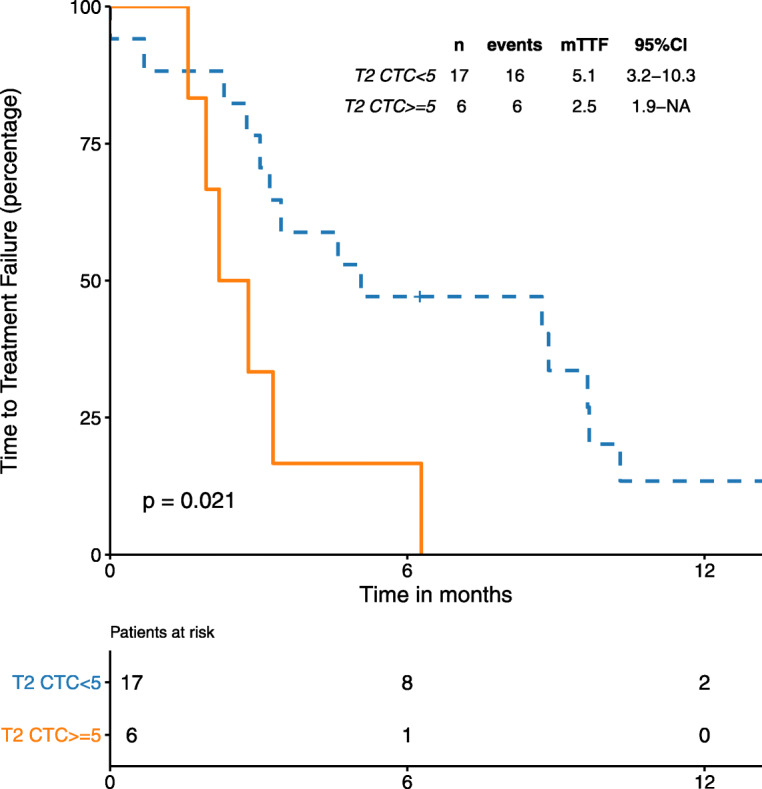
Post-palbociclib TTF by CTC number in patients treated with chemotherapy. CTCs categorized by < 5 and ≥ 5 CTCs cutoff

We correlated CTC counts with TTF for treatments received after exiting the TREnd trial for progressive disease. CTC counts at T2 were available for 34 patients, and for 33 of these patients, post-TREnd treatment clinical data were also available. When patients were analyzed, irrespective of the type of treatment received, we observed no differences in the median TTF (mTTF) according to CTC counts using the 5 CTCs cut-off or CTCs as a continuous variable (mTTF 4.6 months in patients with < 5 CTCs versus 3.0 months in those with ≥ 5 CTCs; *p* = 0.34, *p* = 0.52 for the continuous variable). The same analysis was performed in those patients who received chemotherapy as immediate post-TREnd therapy (*n* = 23). In this subset of patients, those with ≥ 5 CTCs showed significantly lower mTTF than patients with < 5 CTCs (2.5 months versus 5.1 months, respectively, *p* = 0.021) (Fig. 4). Results were consistent when considering CTC counts as a continuous variable (*p* = 0.028). Similar results, although not statistically significant, were seen when using one or more CTCs as a cut-off, both in the entire population, and when considering only patients who received chemotherapy (mTTF 7.4 months in = 0 CTC versus 3.3 months in one or more CTCs *p* = 0.79; mTTF 5.1 months in = 0 CTC versus 3.4 months in one or more CTCs, *p* = 0.87, respectively). mTTF in the subset of patients treated with endocrine therapy as immediate post-TREnd therapy was not analyzed due to the small sample size (*n* = 10). Of note, TTF did not show a significant difference according to treatment type (HR 0.83; 95% CI 0.42, 1.65; *p* = 0.6).

### CTC isolation

Patients with ≥ 5 CTCs after CS enrichment were analyzed for *RB1* expression on CTC. Additional Table 6 shows the number of CTCs sorted by DA. Twenty patients had at least one sample per timepoint with ≥ 5 CTCs corresponding to 37 CS cartridges; 34/37 cartridges were successfully sorted by DA. For 5/20 patients, samples were recovered for all three timepoints, for 7/20 we had samples from two timepoints, and 8/20 patients had a sample from one timepoint only. Overall, 109 samples for downstream analysis were recovered: 50 single CTCs and 59 pools. Whenever possible, we collected both single CTCs and pools at the same timepoint per patient.

### ddPCR assay setup and validation

We set up a duplex ddPCR protocol in order to simultaneously evaluate *RB1* and *GAPDH* using both PDS and PDR cell lines (Additional figure 2). This protocol allowed accurate measurement of palbociclib-induced down-regulation of *RB1* expression in all PDR models compared to parental PDS (T47D PDR versus PDS, *p* < 0.001; MDAMB361 PDR versus PDS, p < 0.001 and BT474 PDR versus PDS, *p* = 0.008) (Additional figure 3A). Sensitivity of the assay was evaluated by performing serial dilutions of the cDNA template. Positive and reliable signals were obtained down to 15 pg of cDNA. The ratio *RB1*/*GAPDH* was maintained across all dilutions (non-significant deviation from zero and from linearity after Fisher test and Runs test) (Additional figure 3B).

### *RB1* and *GAPDH* CTC evaluation by ddPCR and survival analysis

*RB1* and *GAPDH* were successfully evaluated on 10/12 patients at T0, 11/12 patients at T1 and 8/10 patients at T2. Samples with detectable expression of *GAPDH* or *RB1* were considered as CTC-positive, while samples with undetectable expression of both genes (*n* = 34) were considered CTC-negative and discarded. We were able to evaluate 75 samples corresponding to 19/20 patients. Additional Figure 4 shows an example of a CTC enriched by CellSearch, sorted by DEPArray and found to be *RB1*-*GAPDH* positive by ddPCR. Additional Table 7 reports *RB1* and *GAPDH* copy numbers and the numbers of CTCs per patient, per timepoint.

**Fig. 5:**
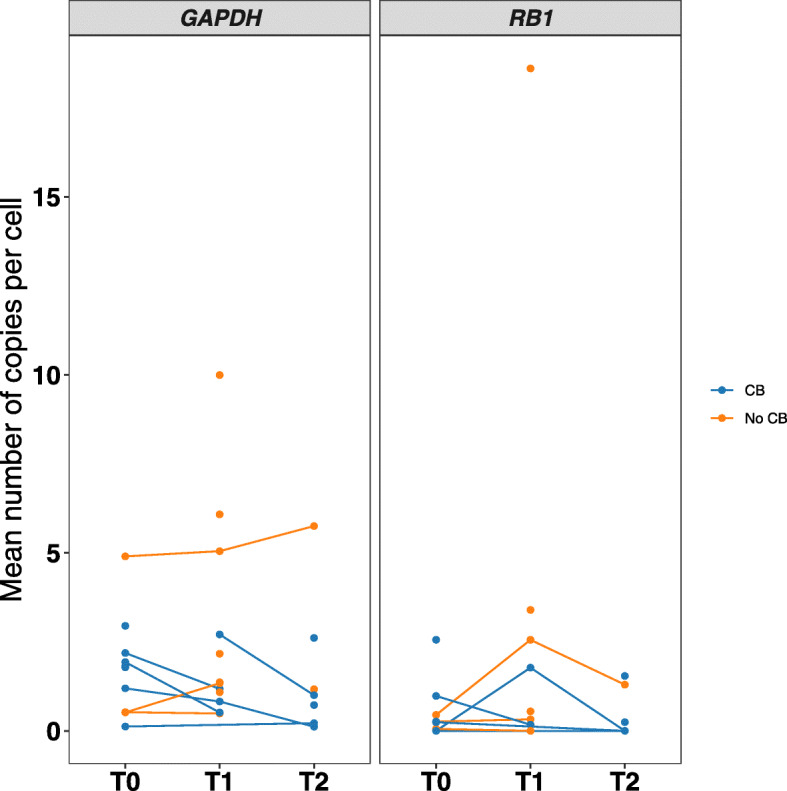
Spaghetti plot of *RB1* and *GAPDH* dynamics*. GAPDH* and *RB1* mean copies number per cell across timepoints according to CB

We found high heterogeneity in the distribution of *RB1* and *GAPDH* expression on single CTCs (Additional Figure 5A). The heterogeneity of both genes is also highlighted by the absence of a linear correlation between CTC number and gene expression levels (*r* = 0.08 *p* = 0.51 for *GAPDH*, *r* = 0.05, *p* = 0.69 for *RB1*). We found a significantly lower mean copy number (MCN) of *RB1* compared to *GAPDH* (*p* = 0.001) (Additional Figure 5B), suggesting low levels of *RB1* expression in most of the cells. No significant modulation of MCN for *RB1* or *GAPDH* was observed across the different timepoints (Additional Figure 6). Figure 5 shows the dynamics of GAPDH and RB1 across timepoints according to CB. Patients with detectable expression of *RB1* in at least one timepoint (*n* = 13) had a longer mPFS compared to those with undetectable levels of *RB1* (*n* = 6) (mPFS 7.6 vs 4.8 months), however, these results were not statistically significant (*p* = 0.49). Additional Table 8 shows the correlation between *RB1* expression and PFS.

## Discussion

The primary aim of this study was to test the use of CTCs as a biomarker of resistance to palbociclib treatment received as a part of the TREnd study. Several meta-analyses have confirmed the presence of CTCs as an independent prognostic factor in numerous metastatic cancer types [4, 19, 20], with patients with a pre-treatment CTC count of 5 per 7.5 mL of whole blood exhibiting a worse PFS and overall survival (OS) [3, 21, 22]. The cutoff of one CTC has been also previously evaluated. A study involving 98 patients with advanced or metastatic breast cancer showed that those with at least one CTC had worse survival compared to those with no CTCs, providing prognostic information in addition to serum tumor markers (CEA and CA 15.3) [23]. Therefore, in this study, we tested both cutoffs (one or more CTCs/7.5 mL of blood and ≥ 5 CTCs/7.5 mL of blood).

In the cTREnd cohort, baseline CTC count was not prognostic, independent of the analyzed cutoff. Interestingly, higher CTC counts were associated with a worse performance status, but despite this, patients with a positive CTC count at baseline did not show worse survival. We cannot exclude that these negative results may be attributable to the small sample size of the present cohort. However, we have previously demonstrated in an equally small cohort of patients treated with endocrine therapy (*n* = 29), that CTC positivity is a strong negative prognostic factor [24]. Therefore, a possible hypothesis could be that palbociclib treatment is able to ameliorate the negative prognostic impact of CTCs in patients with ABC. However, larger studies are needed to validate our results. As CTCs have been used to monitor the efficacy of systemic therapy [1]**,** we collected blood samples after 1 month from the beginning of palbociclib treatment and found that the presence of at least one CTC/7.5 mL of blood identified patients with a significantly worse prognosis. In breast cancer, it has been previously demonstrated a shorter PFS in patients with five or more CTCs detectable at 3 to 5 weeks or at 7 to 9 weeks after the start of chemotherapy or endocrine therapy, respectively [25]. Here, we show that evaluation of CTCs during treatment may also be helpful in patients receiving palbociclib. Based on the hypothesis that the dynamics of CTCs during palbociclib treatment may be useful in monitoring response to therapy, we assessed whether changes in CTC counts were associated with survival. Indeed, we found that patients with an increase of at least three CTCs had a worse PFS compared to patients with no increase. Previous studies have highlighted CTC changes as an early indicator of response to therapy [26–28]. However, none of these studies focused on patients with ER-positive, HER2-negative ABC treated with palbociclib. Overall, our data suggest that patients who are less likely to derive benefit from palbociclib may be identified by the presence of at least one CTC or an increase in CTC count in the blood after the first cycle of treatment, rather than the presence of CTCs prior to commencing treatment.

A major unanswered clinical question regards the best treatment after progression on CDK4/6i [29]. This study investigated the potential role of CTC count in predicting outcome on the next line of therapy received after exiting the TREnd trial. We did not detect any difference in terms of TTF in the whole cohort, comprising patients treated with different treatment modalities. Conversely, when considering a more homogenous group of patients who received chemotherapy after palbociclib progression, we observed that those with five or more CTCs showed a statistically significant worse TTF than patient with < 5 CTCs. We could not analyze patients that received other types of treatment (i.e. endocrine therapies), due to the limited sample size. Therefore, we could not test if the poor prognosis associated with the elevated post-treatment CTC count is specific to chemotherapy or if it relates also to other groups.

Several clinical trials are currently evaluating strategies of incorporating CTC analysis in informing clinical therapeutic choices [30]. To date, results are available from the phase III randomized STIC-CTC trial for patients with metastatic ER-positive, HER2-negative breast cancer, which showed the validity of CTC analysis to guide clinical decision-making [31]. Our results may support the use of CTC counts as a tool to stratify patients in whom greater individualized therapy is currently required during and/or after treatment with palbociclib.

Trials are ongoing with the aim to evaluate treatment decisions based not only on CTC presence, but also on their phenotype [30]. Characterizing CTCs at molecular level might increase the clinical utility of CTC counts. In breast cancer, Kwan et al. developed a digital RNA signature combining CTC enrichment with a quantitative ddPCR defining a CTC-score capable of monitoring patient response to therapy [10]. Furthermore, ddPCR evaluation of the expression of AR-V7 and ARv67es in CTCs has been correlated with response to taxane therapy in men with metastatic prostate cancer [32].

Both de novo and acquired resistance to CDK4/6i have been extensively documented by preclinical and clinical studies [12, 33, 34]. In the past, we and others identified loss of RB1 as a potential molecular alteration that drives resistance to palbociclib in vitro and in vivo [12, 16, 33, 35]. Therefore, in c-TREnd, we tested *RB1* expression on CTCs as a biomarker of response to palbociclib treatment. The use of the combination of CS  and DA  to recover pure CTCs suitable for molecular characterization has been demonstrated previously [36–38], but its ability is limited in that it only sorts samples with five or more CTCs/7.5 mL of blood [39]. Another limitation of CTC gene expression analysis is illustrated by the extremely low content of CTC mRNA, estimated to be approximately 10–30 pg for one single cell [40] depending on the cell type and its physiological state. However, by utilizing ultrasensitive ddPCR [10, 17], and cell lines sensitive and resistant to palbociclib, we were able to set up a ddPCR duplex assay for *RB1* and *GAPDH* expression at single cell level. This assay highlighted the heterogeneity in the expression of both *GAPDH* and *RB1*, confirming that CTCs represent a heterogeneous group of tumor cells [8, 41]. Based on preclinical data [16], we expected a reduction of *RB1* levels during palbociclib treatment but did not observe any significant difference across timepoints, which is likely attributable to the small sample size. However, in line with the hypothetical role of *RB1* in determining resistance to palbociclib, we observed that patients with detectable expression of *RB1* seem to have a better, albeit not statistically significant, outcome than those with undetectable levels *RB1.* This data may indicate that patients with *RB1* expression may derive more benefit from palbociclib treatment. However, more evidence is needed to clarify this issue.

Resistance mechanisms to CDK4/6i are multifactorial and not yet fully understood, involving cell cycle alterations such as *CCNE1* overexpression [42], activation of upstream tyrosine kinase receptors and PI3K/MAPK signaling [43, 44], the Hippo pathway [45], *FGFR* [46] and many other signaling pathways [14, 47]. In this study we focused exclusively on *RB1* expression. Unfortunately, the material isolated from CTCs was not sufficient to allow for a broader analysis of additional biomarkers of CDK4/6i resistance in this study.

Limitations of this study include the small sample size, the exploratory nature of the analyses, and the use of an arbitrary cutoff to determine the increase in CTC number during treatment. Also, patients within TREnd were treated with palbociclib monotherapy or with the endocrine therapy to which they had progressed in the previous line of treatment. More studies are needed to understand if comparable results might be obtained in other clinical settings, including first line in combination with aromatase inhibitors. However, to the best of our knowledge, this is the first study investigating the association between CTC count or gene expression and survival in patients treated with palbociclib.

## Conclusions

In this work, we show that baseline CTC count does not seem to be prognostic in a palbociclib-treated population, but an enumeration of CTCs during treatment can be used as a marker to identify patients with early treatment resistance. We also show that CTC counts at the time of progression may give useful prognostic information for post-palbociclib outcome, supporting further studies on the role of CTCs to guide treatment decisions in this setting. Finally, gene expression analysis on CTCs has proven to be feasible and could provide additional relevant information for personalized therapeutic decisions. Further studies on a larger number of patients are required to validate the current results.

## Supplementary Information

**Additional file 1: Figure S1.** CTCs dynamics. Spider plot of CTC dynamics for each timepoint colored according to CB.

**Additional file 2: Figure S2.** T47D set up of ddPCR assay. A, B-2D plot of T47D PDS and PDR, C, D- concentration plot of T47D PDS and PDR; PDS: sensitive to palbociclib, PDR resistant to palbociclib.

**Additional file 3: Figure S3.** Cell lines set up of ddPCR assay. A) *RB1* expression on PDS and PDR cell lines confirm the reduction of *RB1* expression among PDS and PDS cell lines, *** *p* < 0.001, ** *p* = 0.009, B) linearity of *RB1/GAPDH* ratio from 250 pg up 15 pg of cDNA.

**Additional file 4: Figure S4.** Images of a patient sample CTCs. Images of CTCs identified by A) Cell Search system, B) DEP array. C) 2-D plot *RB1-* droplets positive for *Rb1* expression*, GAPDH-* droplets positive for *GAPDH; RB1+ GAPDH-* droplets positive for both *RB1* and *GAPDH.*

**Additional file 5: Figure S5.** *RB1* and *GAPDH* gene expression analysis. A) distribution of *GAPDH* and *RB1* copies number in single CTCs, B) distribution of mean number of copies of *GAPDH* and *RB1* (tot n copies/n cells).

**Additional file 6: Figure S6.** Mean number of copies of *GAPDH* and *RB1* broken into timepoint.

**Additional file 7: Table S1.** Comparison between baseline patient characteristics of the c-TREnd subpopulation and overall TREnd population.

**Additional file 8: Table S2.** Patients characteristics of c-TREnd cohort, according to treatment arms.

**Additional file 9: Table S3.** Patients’ number of CTCs isolated by CellSearch.

**Additional file 10: Table S4.** Patients characteristics according to baseline CTCs number count.

**Additional file 11: Table S5.** Associations between clinical benefit and CTCs number count.

**Additional file 12: Table S6.** Number of CTCs sorted by DEPArray system.

**Additional file 13: Table S7.** *RB1* and *GAPDH* copy number per patient per timepoint.

**Additional file 14: Table S8.** PFS of patients with *RB1* positive CTC.

## Data Availability

ddPCR gene-expression data for *RB1* and *GAPDH* are available upon reasonable request to the corresponding author.

## Abbreviations

CTCs: Circulating tumor cells
ABC: Advanced breast cancer
ctDNA: Circulating tumor DNA
CDK4/6i: CDK4/6 inhibitors
PFS: Progression-free survival
CB: Clinical benefit
TTF: Time to treatment failure
OS: Overall survival
CR: Complete response
PR: Partial response
SD: Stable disease
HRs: Hazard ratios
CIs: Confidence intervals
PDS: Palbociclib sensitive
PDR: Palbociclib resistant
CS: CellSearch
DA: DEPArray system®
ddPCR: Droplet digital PCR
*GAPDH*: Glyceraldehyde 3-phosphate dehydrogenase
*RB1*: Retinoblastoma 1

## References

[1] Malone ER, Oliva M, Sabatini PJB, Stockley TL, Siu LL (2020). Molecular profiling for precision cancer therapies. Genome Med.

[2] Lianidou E, Pantel K (2019). Liquid biopsies. Genes Chromosom Cancer.

[3] Cristofanilli M, Budd GT, Ellis MJ, Stopeck A, Matera J, Miller MC (2004). Circulating tumor cells, disease progression, and survival in metastatic breast cancer. N Engl J Med.

[4] Bidard F-C, Peeters DJ, Fehm T, Nolé F, Gisbert-Criado R, Mavroudis D, Grisanti S, Generali D, Garcia-Saenz JA, Stebbing J, Caldas C, Gazzaniga P, Manso L, Zamarchi R, de Lascoiti AF, de Mattos-Arruda L, Ignatiadis M, Lebofsky R, van Laere SJ, Meier-Stiegen F, Sandri MT, Vidal-Martinez J, Politaki E, Consoli F, Bottini A, Diaz-Rubio E, Krell J, Dawson SJ, Raimondi C, Rutten A, Janni W, Munzone E, Carañana V, Agelaki S, Almici C, Dirix L, Solomayer EF, Zorzino L, Johannes H, Reis-Filho JS, Pantel K, Pierga JY, Michiels S (2014). Clinical validity of circulating tumour cells in patients with metastatic breast cancer: a pooled analysis of individual patient data. Lancet Oncol.

[5] Cabel L, Proudhon C, Gortais H, Loirat D, Coussy F, Pierga J-Y, Bidard FC (2017). Circulating tumor cells: clinical validity and utility. Int J Clin Oncol.

[6] Pantel K, Speicher MR (2016). The biology of circulating tumor cells. Oncogene.

[7] Castro-Giner F, Aceto N (2020). Tracking cancer progression: from circulating tumor cells to metastasis. Genome Med.

[8] Keller L, Pantel K (2019). Unravelling tumour heterogeneity by single-cell profiling of circulating tumour cells. Nat Rev Cancer.

[9] Aceto N, Bardia A, Wittner BS, Donaldson MC, O’Keefe R, Engstrom A (2018). AR expression in breast cancer CTCs associates with bone metastases. Mol Cancer Res MCR.

[10] Kwan TT, Bardia A, Spring LM, Giobbie-Hurder A, Kalinich M, Dubash T, Sundaresan T, Hong X, LiCausi JA, Ho U, Silva EJ, Wittner BS, Sequist LV, Kapur R, Miyamoto DT, Toner M, Haber DA, Maheswaran S (2018). A digital RNA signature of circulating tumor cells predicting early therapeutic response in localized and metastatic breast cancer. Cancer Discov.

[11] Miyamoto DT, Lee RJ, Kalinich M, LiCausi JA, Zheng Y, Chen T (2018). An RNA-based digital circulating tumor cell signature is predictive of drug response and early dissemination in prostate cancer. Cancer Discov.

[12] Guarducci C, Bonechi M, Boccalini G, Benelli M, Risi E, Di Leo A (2017). Mechanisms of resistance to CDK4/6 inhibitors in breast cancer and potential biomarkers of response. Breast Care Basel Switz.

[13] Condorelli R, Spring L, O’Shaughnessy J, Lacroix L, Bailleux C, Scott V (2018). Polyclonal RB1 mutations and acquired resistance to CDK 4/6 inhibitors in patients with metastatic breast cancer. Ann Oncol Off J Eur Soc Med Oncol.

[14] Migliaccio I, Bonechi M, McCartney A, Guarducci C, Benelli M, Biganzoli L (2020). CDK4/6 inhibitors: a focus on biomarkers of response and post-treatment therapeutic strategies in hormone receptor-positive HER2-negative breast cancer. Cancer Treat Rev.

[15] Malorni L, Curigliano G, Minisini AM, Cinieri S, Tondini CA, D’Hollander K (2018). Palbociclib as single agent or in combination with the endocrine therapy received before disease progression for estrogen receptor-positive, HER2-negative metastatic breast cancer: TREnd trial. Ann Oncol Off J Eur Soc Med Oncol.

[16] Guarducci C, Bonechi M, Benelli M, Biagioni C, Boccalini G, Romagnoli D, Verardo R, Schiff R, Osborne CK, de Angelis C, di Leo A, Malorni L, Migliaccio I (2018). Cyclin E1 and Rb modulation as common events at time of resistance to palbociclib in hormone receptor-positive breast cancer. NPJ Breast Cancer.

[17] Ma Y, Luk A, Young FP, Lynch D, Chua W, Balakrishnar B, et al. Droplet digital PCR based androgen receptor variant 7 (AR-V7) detection from prostate cancer patient blood biopsies. Int J Mol Sci. 2016;17(8). 10.3390/ijms17081264 PMC5000662 27527157

[18] Dehm SM, Montgomery B, Plymate SR (2019). AR-variant-positive CTC: a surrogate for a surrogate for taxane therapy outcome?. Clin Cancer Res Off J Am Assoc Cancer Res.

[19] Huang X, Gao P, Song Y, Sun J, Chen X, Zhao J, et al. Meta-analysis of the prognostic value of circulating tumor cells detected with the CellSearch system in colorectal cancer. BMC Cancer. 2015;15 Disponibile su: https://www.ncbi.nlm.nih.gov/pmc/articles/PMC4389311/. [citato 19 aprile 2020]. 10.1186/s12885-015-1218-9 PMC4389311 25880692

[20] Yang C, Zou K, Yuan Z, Guo T, Xiong B (2018). Prognostic value of circulating tumor cells detected with the CellSearch system in patients with gastric cancer: evidence from a meta-analysis. OncoTargets Ther.

[21] Pierga J-Y, Hajage D, Bachelot T, Delaloge S, Brain E, Campone M, Diéras V, Rolland E, Mignot L, Mathiot C, Bidard FC (2012). High independent prognostic and predictive value of circulating tumor cells compared with serum tumor markers in a large prospective trial in first-line chemotherapy for metastatic breast cancer patients. Ann Oncol Off J Eur Soc Med Oncol.

[22] Cristofanilli M, Hayes DF, Budd GT, Ellis MJ, Stopeck A, Reuben JM (2005). Circulating tumor cells: a novel prognostic factor for newly diagnosed metastatic breast cancer. J Clin Oncol Off J Am Soc Clin Oncol.

[23] Shiomi-Mouri Y, Kousaka J, Ando T, Tetsuka R, Nakano S, Yoshida M, Fujii K, Akizuki M, Imai T, Fukutomi T, Kobayashi K (2016). Clinical significance of circulating tumor cells (CTCs) with respect to optimal cut-off value and tumor markers in advanced/metastatic breast cancer. Breast Cancer Tokyo Jpn.

[24] Bonechi M, Galardi F, Biagioni C, De Luca F, Bergqvist M, Neumüller M (2018). Plasma thymidine kinase-1 activity predicts outcome in patients with hormone receptor positive and HER2 negative metastatic breast cancer treated with endocrine therapy. Oncotarget.

[25] Liu MC, Shields PG, Warren RD, Cohen P, Wilkinson M, Ottaviano YL (2009). Circulating tumor cells: a useful predictor of treatment efficacy in metastatic breast cancer. J Clin Oncol Off J Am Soc Clin Oncol.

[26] Nakamura S, Yagata H, Ohno S, Yamaguchi H, Iwata H, Tsunoda N, Ito Y, Tokudome N, Toi M, Kuroi K, Suzuki E (2010). Multi-center study evaluating circulating tumor cells as a surrogate for response to treatment and overall survival in metastatic breast cancer. Breast Cancer Tokyo Jpn.

[27] Hartkopf AD, Wagner P, Wallwiener D, Fehm T, Rothmund R (2011). Changing levels of circulating tumor cells in monitoring chemotherapy response in patients with metastatic breast cancer. Anticancer Res.

[28] Deutsch TM, Stefanovic S, Feisst M, Fischer C, Riedel F, Fremd C, et al. Cut-off analysis of CTC change under systemic therapy for defining early therapy response in metastatic breast cancer. Cancers. 2020;12(4). 10.3390/cancers12041055 PMC7226373 32344685

[29] Rossi L, Biagioni C, McCartney A, Migliaccio I, Curigliano G, Sanna G (2019). Clinical outcomes after palbociclib with or without endocrine therapy in postmenopausal women with hormone receptor positive and HER2-negative metastatic breast cancer enrolled in the TREnd trial. Breast Cancer Res BCR.

[30] Schochter F, Friedl TWP, de Gregorio A, Krause S, Huober J, Rack B, et al. Are circulating tumor cells (CTCs) ready for clinical use in breast cancer? An overview of completed and ongoing trials using CTCs for clinical treatment decisions. Cells. 2019;8(11). 10.3390/cells8111412 PMC6912467 31717458

[31] Bidard F-C, Jacot W, Kiavue N, Dureau S, Kadi A, Brain E (2021). Efficacy of circulating tumor cell count-driven vs clinician-driven first-line therapy choice in hormone receptor-positive, ERBB2-negative metastatic breast cancer: the STIC CTC randomized clinical trial. JAMA Oncol.

[32] Tagawa ST, Antonarakis ES, Gjyrezi A, Galletti G, Kim S, Worroll D (2019). Expression of AR-V7 and ARv567es in circulating tumor cells correlates with outcomes to taxane therapy in men with metastatic prostate cancer treated in TAXYNERGY. Clin Cancer Res Off J Am Assoc Cancer Res.

[33] McCartney A, Migliaccio I, Bonechi M, Biagioni C, Romagnoli D, De Luca F (2019). Mechanisms of resistance to CDK4/6 inhibitors: potential implications and biomarkers for clinical practice. Front Oncol.

[34] Pandey K, An H-J, Kim SK, Lee SA, Kim S, Lim SM (2019). Molecular mechanisms of resistance to CDK4/6 inhibitors in breast cancer: a review. Int J Cancer.

[35] Herrera-Abreu MT, Palafox M, Asghar U, Rivas MA, Cutts RJ, Garcia-Murillas I (2016). Early adaptation and acquired resistance to CDK4/6 inhibition in estrogen receptor-positive breast cancer. Cancer Res.

[36] Peeters DJE, De Laere B, Van den Eynden GG, Van Laere SJ, Rothé F, Ignatiadis M (2013). Semiautomated isolation and molecular characterisation of single or highly purified tumour cells from CellSearch enriched blood samples using dielectrophoretic cell sorting. Br J Cancer.

[37] Fabbri F, Carloni S, Zoli W, Ulivi P, Gallerani G, Fici P (2013). Detection and recovery of circulating colon cancer cells using a dielectrophoresis-based device: KRAS mutation status in pure CTCs. Cancer Lett.

[38] Salvianti F, Rotunno G, Galardi F, De Luca F, Pestrin M, Vannucchi AM (2015). Feasibility of a workflow for the molecular characterization of single cells by next generation sequencing. Biomol Detect Quantif.

[39] De Luca F, Rotunno G, Salvianti F, Galardi F, Pestrin M, Gabellini S (2016). Mutational analysis of single circulating tumor cells by next generation sequencing in metastatic breast cancer. Oncotarget.

[40] Molecular Cloning: A Laboratory Manual, 3rd ed., Vols 1,2 and 3 J.F. Sambrook and D.W. Russell, ed., Cold Spring Harbor Laboratory Press, 2001, 2100 pp., soft cover | Sigma-Aldrich [Internet]. 2020 [citato 19 aprile 2020]. Disponibile su: https://www.sigmaaldrich.com/catalog/product/sigma/m8265?lang=it&region=IT

[41] Pestrin M, Salvianti F, Galardi F, De Luca F, Turner N, Malorni L (2015). Heterogeneity of PIK3CA mutational status at the single cell level in circulating tumor cells from metastatic breast cancer patients. Mol Oncol.

[42] Turner NC, Liu Y, Zhu Z, Loi S, Colleoni M, Loibl S (2019). Cyclin E1 expression and palbociclib efficacy in previously treated hormone receptor-positive metastatic breast cancer. J Clin Oncol Off J Am Soc Clin Oncol.

[43] Jansen VM, Bhola NE, Bauer JA, Formisano L, Lee K-M, Hutchinson KE (2017). Kinome-wide RNA interference screen reveals a role for PDK1 in acquired resistance to CDK4/6 inhibition in ER-positive breast cancer. Cancer Res.

[44] Costa C, Wang Y, Ly A, Hosono Y, Murchie E, Walmsley CS, Huynh T, Healy C, Peterson R, Yanase S, Jakubik CT, Henderson LE, Damon LJ, Timonina D, Sanidas I, Pinto CJ, Mino-Kenudson M, Stone JR, Dyson NJ, Ellisen LW, Bardia A, Ebi H, Benes CH, Engelman JA, Juric D (2020). PTEN loss mediates clinical cross-resistance to CDK4/6 and PI3Kα inhibitors in breast cancer. Cancer Discov.

[45] Li Z, Razavi P, Li Q, Toy W, Liu B, Ping C (2018). Loss of the FAT1 tumor suppressor promotes resistance to CDK4/6 inhibitors via the hippo pathway. Cancer Cell.

[46] Formisano L, Lu Y, Servetto A, Hanker AB, Jansen VM, Bauer JA (2019). Aberrant FGFR signaling mediates resistance to CDK4/6 inhibitors in ER+ breast cancer. Nat Commun.

[47] Wander SA, Cohen O, Gong X, Johnson GN, Buendia-Buendia JE, Lloyd MR, Kim D, Luo F, Mao P, Helvie K, Kowalski KJ, Nayar U, Waks AG, Parsons SH, Martinez R, Litchfield LM, Ye XS, Yu C, Jansen VM, Stille JR, Smith PS, Oakley GJ, Chu QS, Batist G, Hughes ME, Kremer JD, Garraway LA, Winer EP, Tolaney SM, Lin NU, Buchanan SG, Wagle N (2020). The genomic landscape of intrinsic and acquired resistance to cyclin-dependent kinase 4/6 inhibitors in patients with hormone receptor-positive metastatic breast cancer. Cancer Discov.

