# Supplementary figures and images for: Circulating tumor cells and palbociclib treatment in patients with ER-positive, HER2-negative advanced breast cancer: results from a translational sub-study of the TREnd trial
Source: Breast Cancer Res. 2021 Mar 24;23:38. doi: 10.1186/s13058-021-01415-w (PMC7992319; doi:10.1186/s13058-021-01415-w)

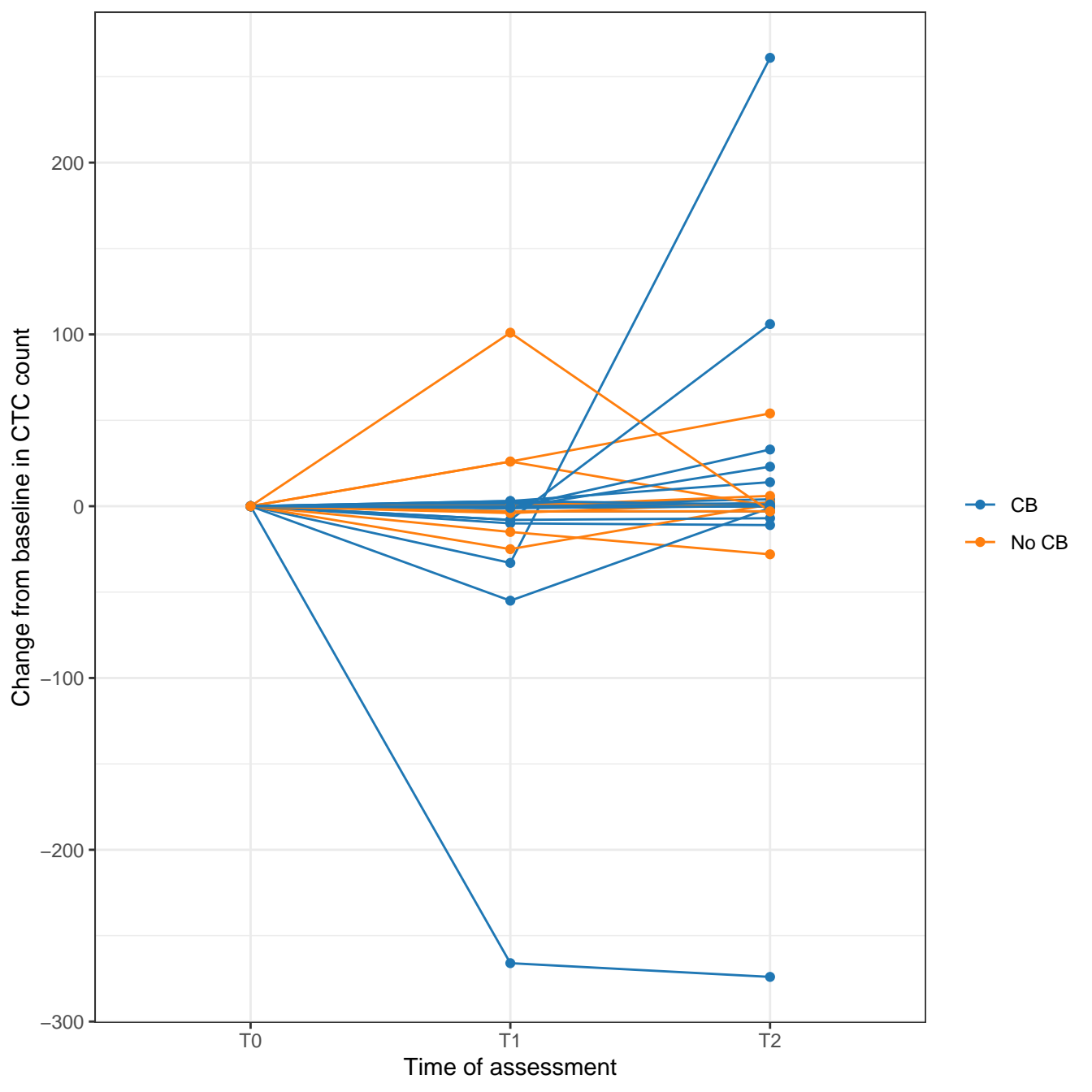

Supplement: Supplementary file 1 — Additional file 1: Figure S1. CTCs dynamics. Spider plot of CTC dynamics for each timepoint colored according to CB. [file 13058_2021_1415_MOESM1_ESM.pdf]

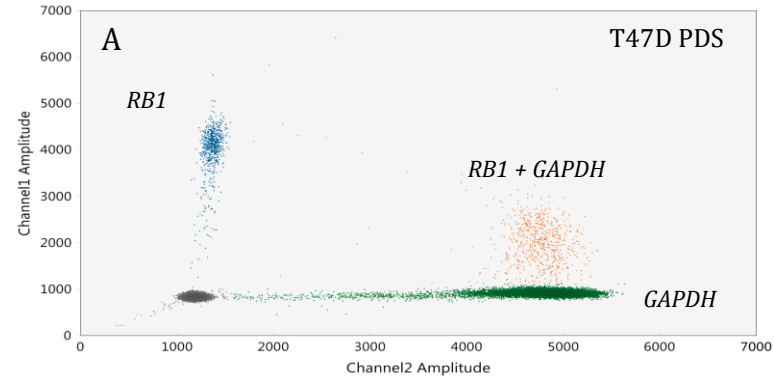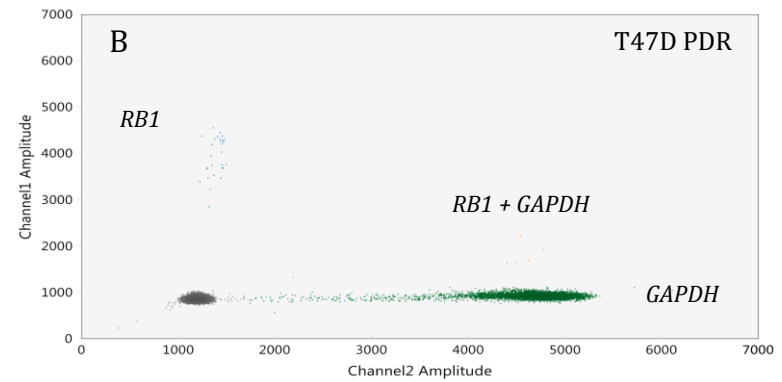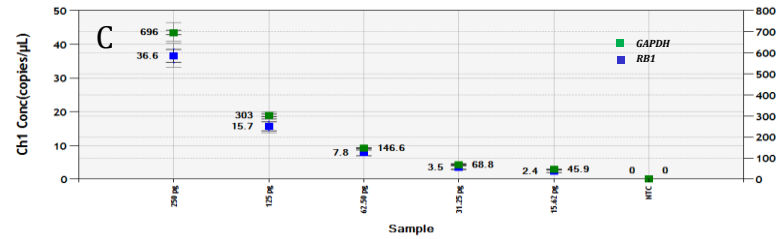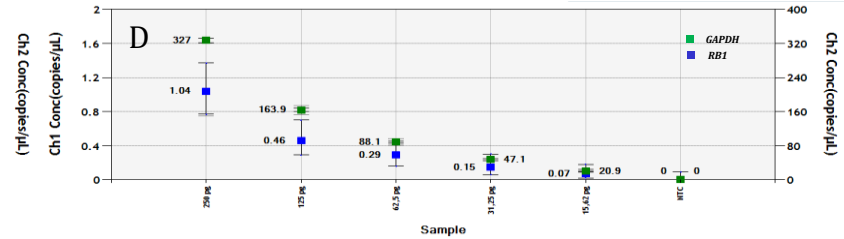

Supplement: Supplementary file 2 — Additional file 2: Figure S2. T47D set up of ddPCR assay. A, B-2D plot of T47D PDS and PDR, C, D- concentration plot of T47D PDS and PDR; PDS: sensitive to palbociclib, PDR resistant to palbociclib. [file 13058_2021_1415_MOESM2_ESM.pdf]

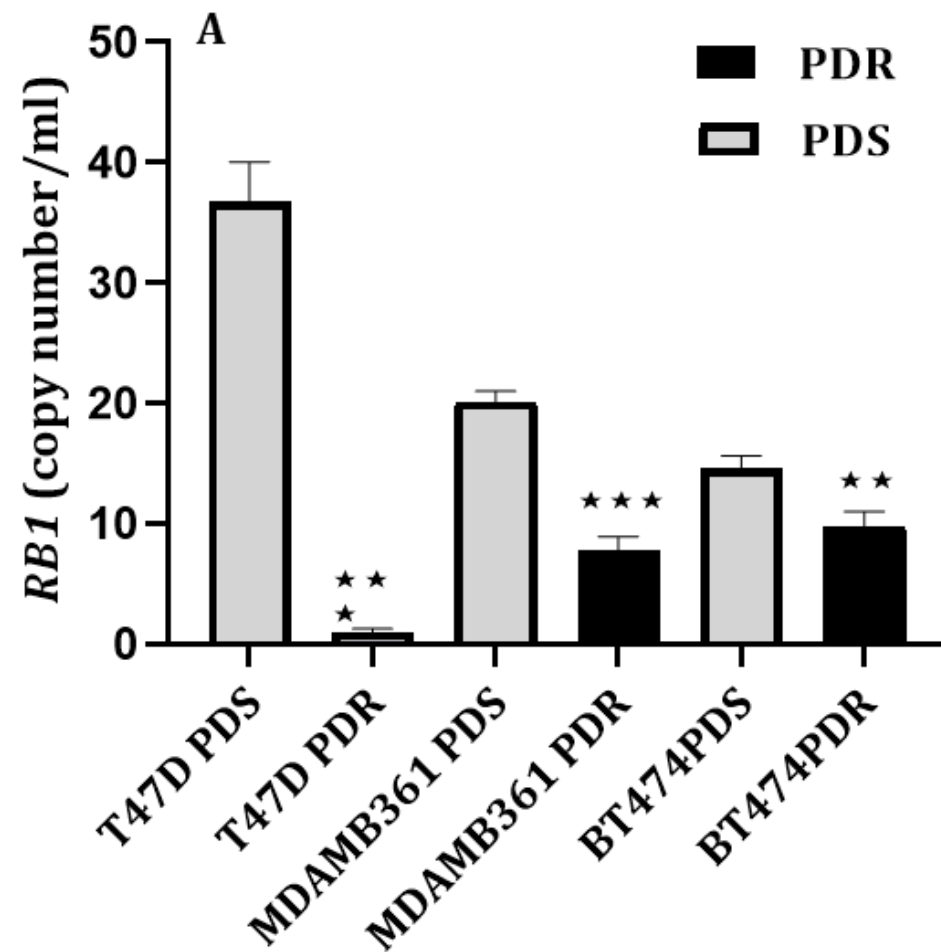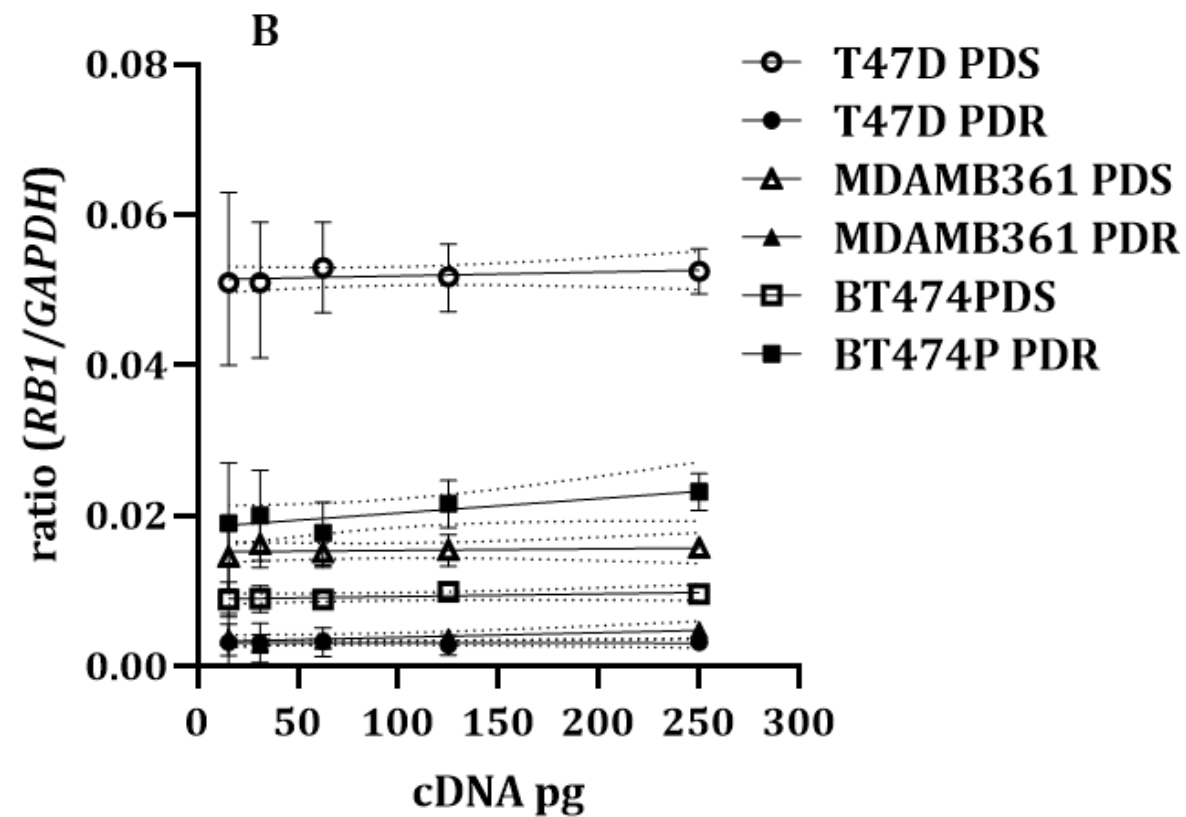

Supplement: Supplementary file 3 — Additional file 3: Figure S3. Cell lines set up of ddPCR assay. A) RB1 expression on PDS and PDR cell lines confirm the reduction of RB1 expression among PDS and PDS cell lines, *** p < 0.001, ** p = 0.009, B) linearity of RB1/GAPDH ratio from 250 pg up 15 pg of cDNA. [file 13058_2021_1415_MOESM3_ESM.pdf]

**A**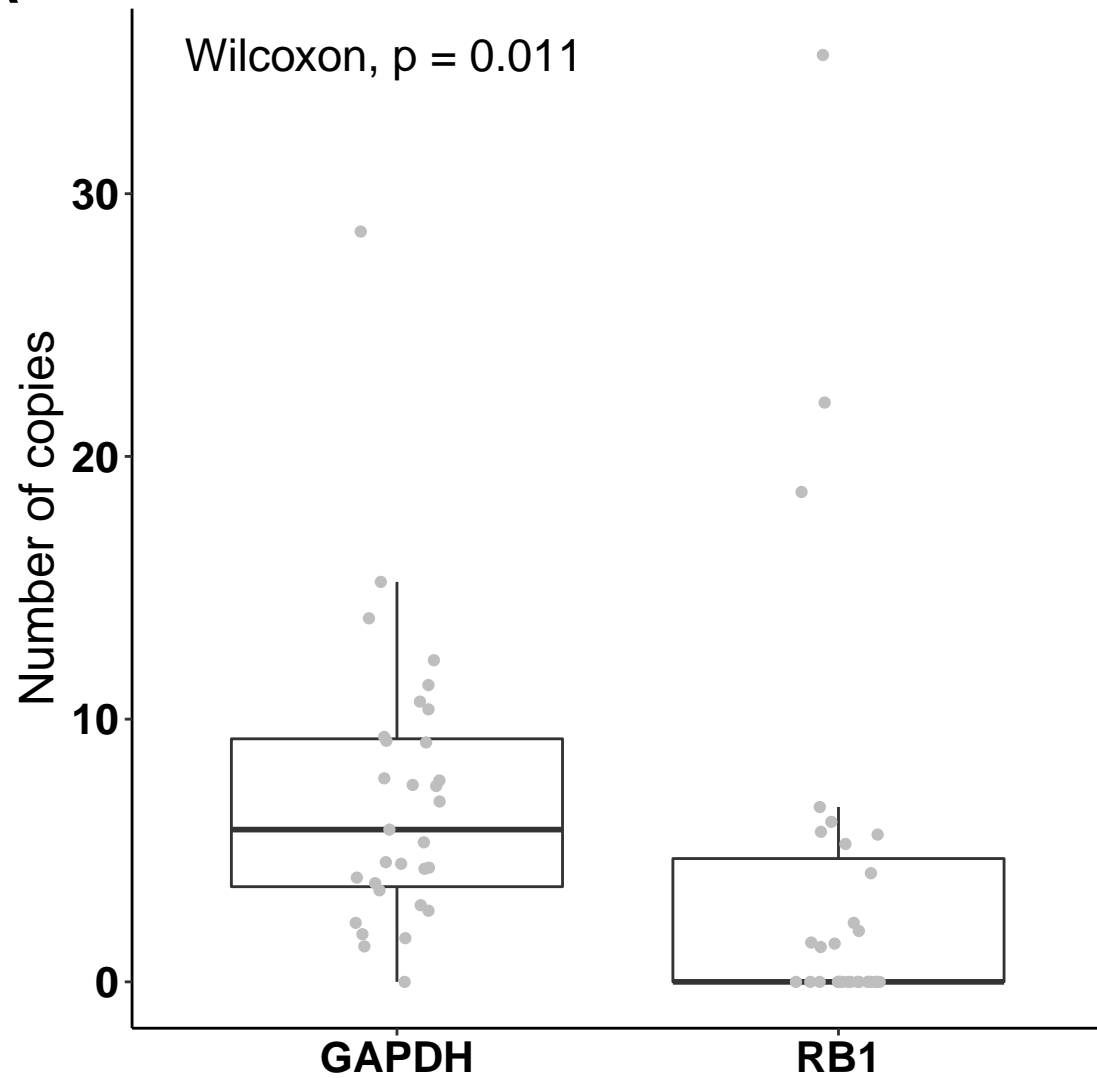**B**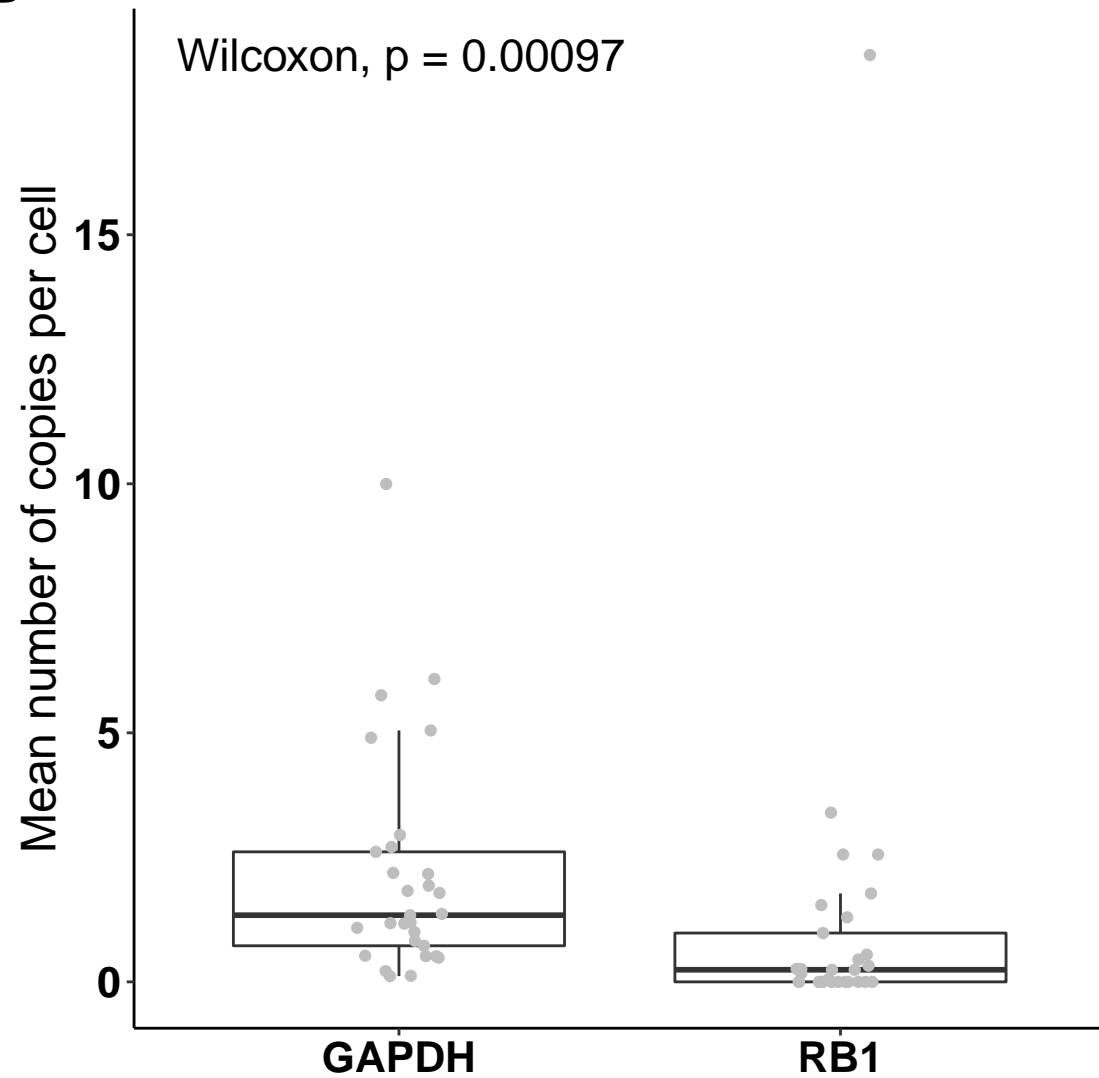

Supplement: Supplementary file 5 — Additional file 5: Figure S5. RB1 and GAPDH gene expression analysis. A) distribution of GAPDH and RB1 copies number in single CTCs, B) distribution of mean number of copies of GAPDH and RB1 (tot n copies/n cells). [file 13058_2021_1415_MOESM5_ESM.pdf]

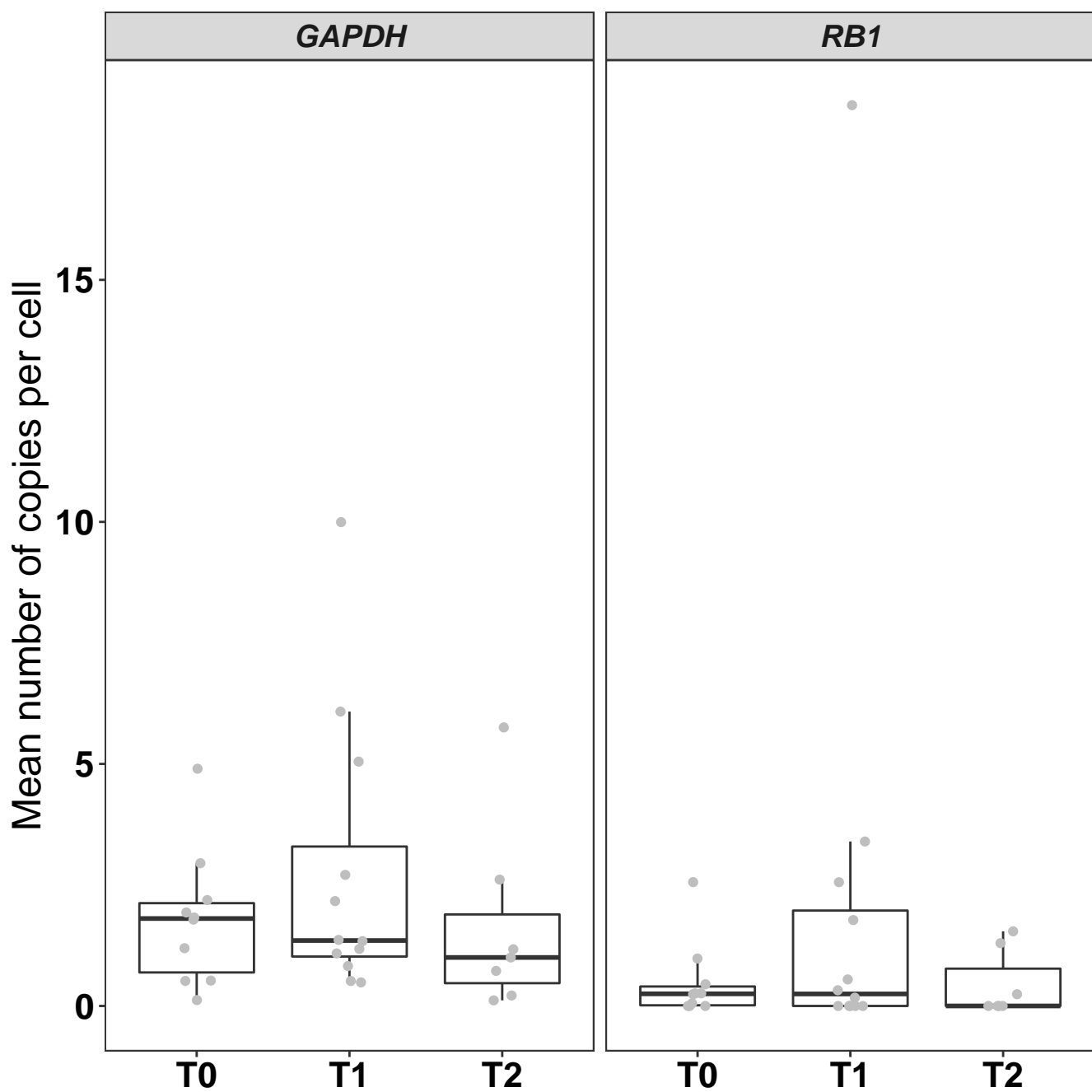

Supplement: Supplementary file 6 — Additional file 6: Figure S6. Mean number of copies of GAPDH and RB1 broken into timepoint. [file 13058_2021_1415_MOESM6_ESM.pdf]
