# Supplementary material for: Circulating tumor cells and palbociclib treatment in patients with ER-positive, HER2-negative advanced breast cancer: results from a translational sub-study of the TREnd trial
Source: Breast Cancer Res. 2021 Mar 24;23:38. doi: 10.1186/s13058-021-01415-w (PMC7992319; doi:10.1186/s13058-021-01415-w)
Supplement: Supplementary file 4 — Additional file 4: Figure S4. Images of a patient sample CTCs. Images of CTCs identified by A) Cell Search system, B) DEP array. C) 2-D plot RB1- droplets positive for Rb1 expression, GAPDH- droplets positive for GAPDH; RB1+ GAPDH- droplets positive for both RB1 and GAPDH. [file 13058_2021_1415_MOESM4_ESM.pdf]

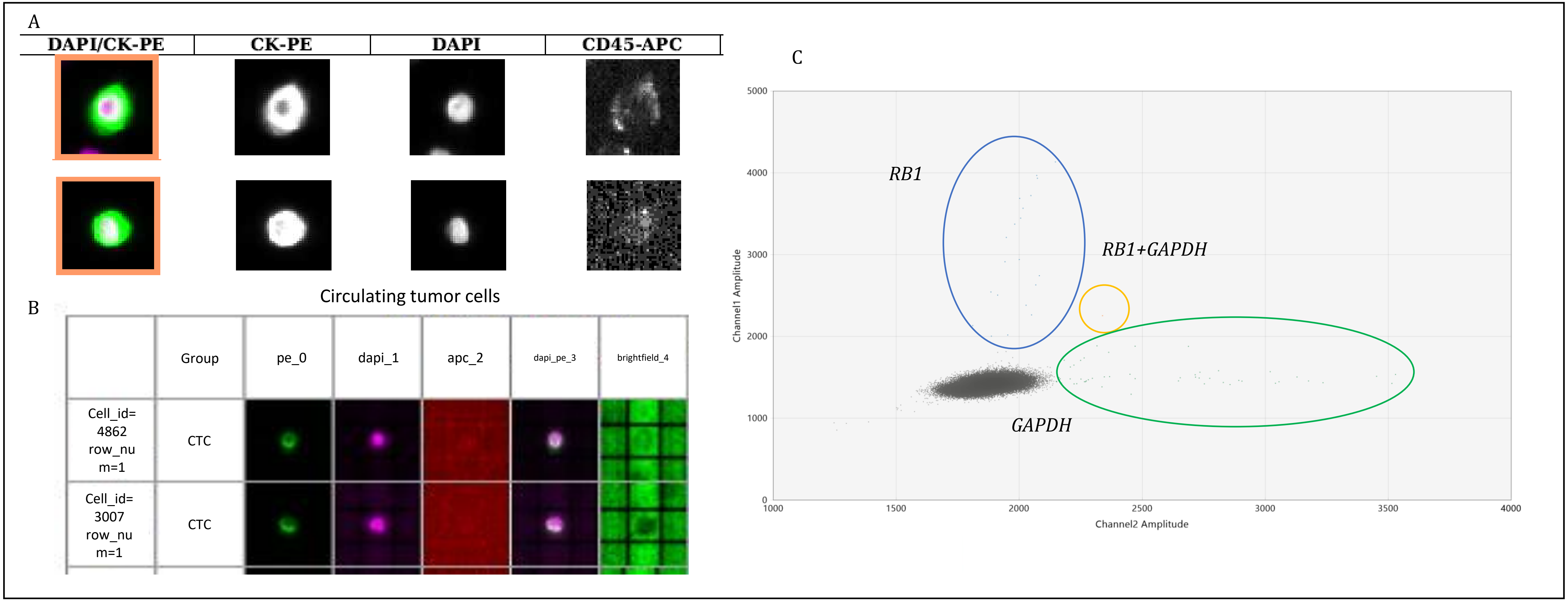
A
DAPI/CK-PE
CK-PE
DAPI
CD45-APC
B
Circulating tumor cells
Group
pe_0
dapi_1
apc_2
dapi_pe_3
brightfield_4
Cell_id= 4862 row_num=1
CTC
Cell_id= 3007 row_num=1
CTC
C
RB1
RB1+GAPDH
GAPDH
Channel1 Amplitude
Channel2 Amplitude
0
1000
2000
3000
4000
5000
1000
1500
2000
2500
3000
3500
4000
